# Will a “multivitamin” a day keep the “MASLD doctor” away?

**DOI:** 10.20517/mtod.2025.24

**Published:** 2025-09-11

**Authors:** Fernando Bril

**Affiliations:** 1 Division of Endocrinology, Diabetes and Metabolism, Department of Medicine, University of Alabama at Birmingham, Birmingham, AL 35233, USA.; 2 UAB Comprehensive Diabetes Center, University of Alabama at Birmingham, Birmingham, AL 35233, USA.; 3 Division of Endocrinology, Diabetes and Metabolism, Department of Medicine, Birmingham VA Medical Center, Birmingham, AL 35233, USA.

**Keywords:** Metabolic dysfunction-associated steatotic liver disease, steatohepatitis, cofactors, one-carbon metabolism, MASH

## Abstract

This commentary discusses the results of a study that assessed the relationship between homocysteine metabolism and histological severity of metabolic dysfunction-associated steatotic liver disease (MASLD), and applied a mathematical model to examine how replacement with different cofactors (pyridoxine, cobalamin, betaine, and folate) may affect homocysteine levels in patients with MASLD. It highlights the clinical implications of the study and examines the pathophysiological support behind the detected associations. It also discusses its limitations, emphasizing the need for further longitudinal and interventional studies to confirm whether modulating homocysteine levels could be a viable therapeutic strategy for MASLD.

I read with interest the study by Suzuki *et al*.^[1]^, which assessed the relationship between homocysteine metabolism and the histological severity of nonalcoholic fatty liver disease (NAFLD), and modeled how replacement with different cofactors, such as pyridoxine (vitamin B6), cobalamin (vitamin B12), betaine, and folic acid (vitamin B9), may affect hepatic homocysteine levels in patients with NAFLD. Not all NAFLD patients included in the study fulfilled the criteria for metabolic dysfunction-associated steatotic liver disease (MASLD); therefore, throughout this commentary, we will continue using the term NAFLD when referring specifically to the study population. We will use MASLD when referring globally to the liver condition. In Figure 1, we have summarized the main metabolic pathways that regulate homocysteine levels. As shown, vitamin B6, vitamin B12, betaine, and folic acid participate in distinct metabolic pathways that affect homocysteine levels, suggesting the potential for additive benefits when combined. It should also be noted that the transsulfuration pathway (leading to cysteine) occurs primarily in the liver, but also in the kidneys, pancreas, and small intestine^[2]^. Remethylation of homocysteine to methionine mediated by betaine occurs in the liver and kidney, whereas the vitamin B12-dependent pathway is present in most tissues^[3]^. While abnormalities in one-carbon metabolism have been associated with MASLD in dietary and genetic animal models of metabolic dysfunction-associated steatohepatitis (MASH)^[4]^, their role in human MASH is less well established, making studies such as that by Suzuki *et al*. particularly valuable^[1]^.

To shed light on this issue, the authors analyzed data from 82 patients with biopsy-proven NAFLD. The study cohort consisted of middle-aged, mostly non-Hispanic White individuals, with a broad range of disease severity, including 79% with nonalcoholic steatohepatitis (NASH) and 46% with advanced fibrosis. However, given the relatively small sample size and the underrepresentation of racial/ethnic minorities, it remains unclear whether the findings can be generalized to other populations. In logistic regression analyses, lower gene expression of cystathionine beta-synthase (CBS) and phosphatidylethanolamine N-methyltransferase (PEMT) was associated with a higher risk of hepatic fibrosis. Furthermore, lower hepatic gene expression of paraoxonase (PON)1 and PON3 correlated with a higher risk of hepatic steatosis.

Given the cross-sectional nature of this part of the study, no causality can be determined, and the implications of these associations are unknown. However, it is interesting to note that all detected associations have a strong pathophysiological support, and they have previously been reported in the literature in other experimental conditions^[5-9]^. For example, lower expression of CBS, a key enzyme of the transsulfuration pathway, would result in elevated homocysteine and methionine levels, and a consequent reduction of glutathione production. In turn, high homocysteine and/or low glutathione have been shown to contribute to hepatic inflammation and liver fibrosis in prior reports^[5-7]^. By decreasing glutathione levels, impaired transsulfuration would increase susceptibility to oxidative stress, which in turn could lead to mitochondrial dysfunction and cellular damage^[10]^. Lower activity of PEMT would result in reduced levels of phosphatidylcholine, which is essential for very-low-density lipoprotein (VLDL) secretion, and its deficiency has been associated with MASLD^[11]^. Moreover, lower PEMT activity could lead to choline deficiency, particularly in choline-deficient diets, which has been associated with liver damage and other organ dysfunction^[12]^. Finally, lower PEMT activity can result in increased RNA and DNA methylation, affecting epigenetic (and epitranscriptomic) regulation^[13]^. This would occur as a consequence of increased S-adenosylmethionine (SAM) availability, with a consequent decrease in S-adenosylhomocysteine (SAH), thereby enhancing the activity of methyltransferases^[14]^. Paraoxonases participate in the metabolism of homocysteine-thiolactone, protecting against N-homocysteinylation of proteins (PON1 > > PON3). Both PON1 and PON3 have also been found to be reduced in patients with MASLD in prior reports^[8,9]^, and PON3 was even reduced in isolated high-density lipoprotein (HDL) lipoproteins from patients with MASLD^[15]^.

The authors went further by applying a mathematical model to predict hepatic homocysteine levels based on consumption of one-carbon metabolism cofactors, such as pyridoxine, cobalamin, betaine, folate, or the combination of the four, along with differences in men and women. Based on these models, they showed that the combination of the four cofactors was able to induce lower levels of hepatic homocysteine compared to each cofactor individually, with some interesting differences in men *vs.* premenopausal women *vs.* postmenopausal women. While we could hypothesize that this may also occur in patients without NAFLD, no controls were included in the study. Most of the evidence of the effects of supplements on homocysteine levels in healthy controls comes from studies looking at serum homocysteine levels, and not hepatic levels as modelled in the study by Suzuki *et al*.^[1,16-20]^. For example, a meta-analysis of individual data from 25 randomized, controlled trials showed a dose-dependent reduction in plasma homocysteine concentrations with increasing folic acid doses^[16]^. Moreover, combination with vitamin B12 was associated with an additional 7% reduction in homocysteine levels in that study. A network meta-analysis including 16 studies reported that combination of folate, vitamin B6, and vitamin B12 was the most favorable to reduce blood homocysteine levels^[20]^. Of note, serum homocysteine levels do not represent liver levels, as other organs (e.g., kidney and skeletal muscle) also play a key role in determining serum homocysteine levels^[21]^. While the findings reported by Suzuki *et al*. are encouraging, it remains to be determined whether reducing hepatic homocysteine or homocysteine-thiolactone would result in an improvement in MASLD/MASH^[1]^. Longitudinal and interventional studies are needed in order to appropriately answer that question.

The idea that nutritional deficiencies could be related to MASLD/MASH is not new^[22]^. Several cross-sectional studies have reported associations between nutritional deficiencies and MASLD/MASH^[23-27]^, although results have not been consistent, and a causal relationship cannot be established. Moreover, different nutrients and vitamins, including some that participate in one-carbon metabolism, have been replaced in randomized, controlled trials in patients with MASH with inconsistent results^[28-31]^.

In summary, the study by Suzuki *et al*. provides an interesting view regarding the potential role of one-carbon metabolism in the development and progression of MASLD in humans^[1]^. However, whether these findings translate into a potential therapeutic target by modulating hepatic homocysteine levels remains to be determined in prospective, interventional studies. Until then, it is unclear whether a ‘multivitamin’ every day would benefit patients with MASLD.

## Figures and Tables

**Figure 1.**
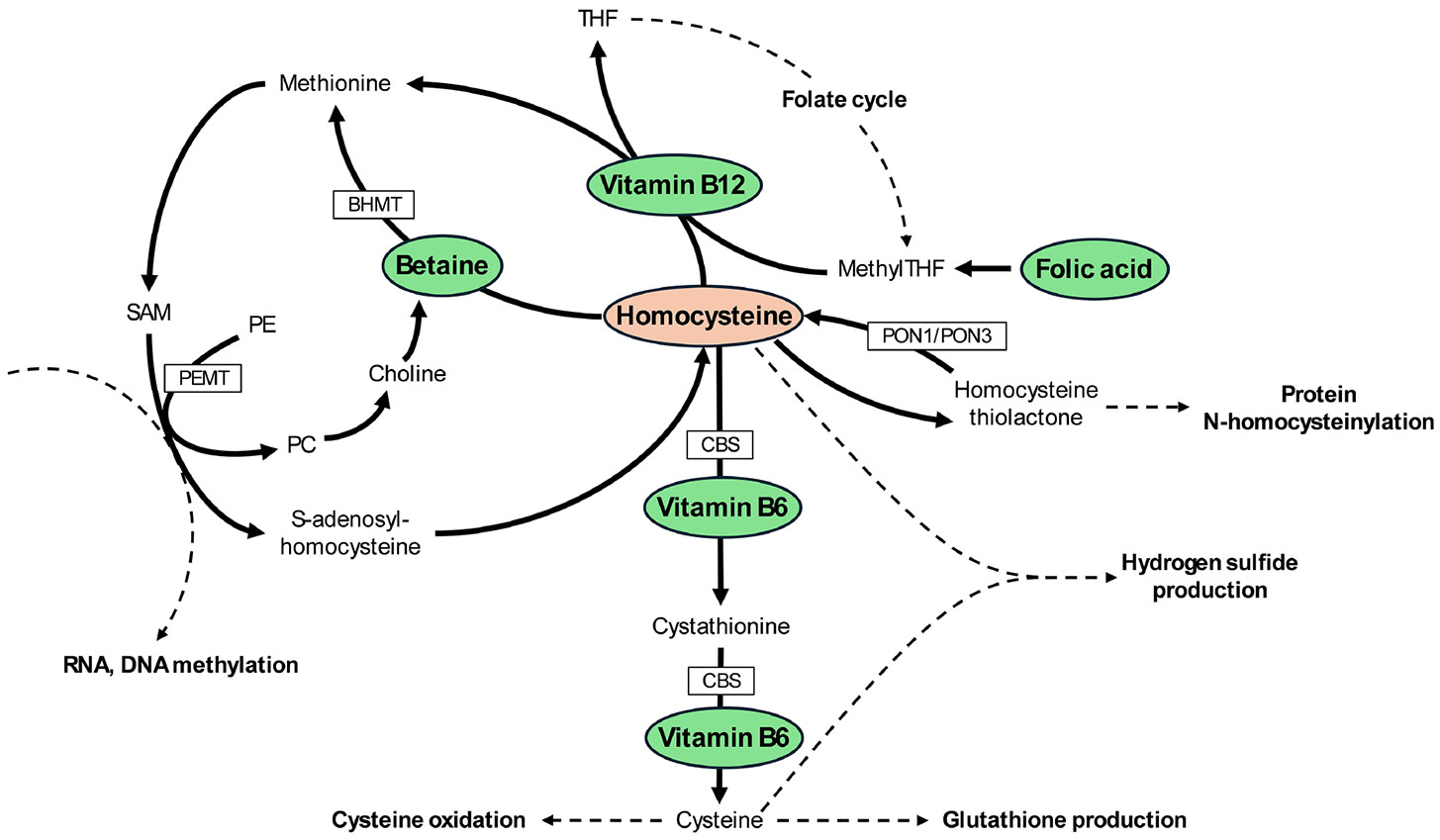
Pathways involved in the metabolism of homocysteine and role of cobalamin (vitamin B12), pyridoxine (vitamin B6), folic acid, and betaine. SAM: S-adenosylmethionine; SAH: S-adenosylhomocysteine; PE: phosphatidylethanolamine; PC: phosphatidylcholine; PEMT: phosphatidylethanolamine N-methyltransferase; BHMT: betaine-homocysteine methyltransferase; CBS: cystathionine β-synthase; THF: tetrahydrofolate; MethylTHF: 5-methyltetrahydrofolate; PON1: paraoxonase 1; PON3: paraoxonase 3.
